# Integrated Analytical Approach to Identify Whey Permeate Powder Caking: Revealing Internal Structure Using X-Ray Micro-Tomography

**DOI:** 10.3390/molecules31101607

**Published:** 2026-05-11

**Authors:** Marek Szołtysik, Nesa Dibagar, Małgorzata Serowik, Monika Słupska, Artur Gryszkin, Adam Figiel

**Affiliations:** 1 Department of Functional Food Products Development, Faculty of Biotechnology and Food Science, Wrocław University of Environmental and Life Sciences, 37a Chełmońskiego Str., 51-630 Wrocław, Poland; marek.szoltysik@upwr.edu.pl; 2 Department of Food Chemistry and Biocatalysis, Faculty of Biotechnology and Food Science, Wrocław University of Environmental and Life Sciences, 25 Norwida Str., 50-375 Wrocław, Poland; 3 Institute of Agricultural Engineering, Wrocław University of Environmental and Life Sciences, 37 Chełmońskiego Str., 51-630 Wrocław, Poland; malgorzata.serowik@upwr.edu.pl (M.S.); adam.figiel@upwr.edu.pl (A.F.); 4 Department of Engineering Fundamentals, Institute of Agricultural Engineering, Wrocław University of Environmental and Life Sciences, 37 Chełmońskiego Str., 51-630 Wrocław, Poland; monika.slupska@upwr.edu.pl; 5 Department of Food Storage and Technology, Faculty of Biotechnology and Food Science, Wrocław University of Environmental and Life Sciences, 37 Chełmońskiego Str., 51-630 Wrocław, Poland; artur.gryszkin@upwr.edu.pl

**Keywords:** dairy-based powder, instability, caking behavior, micro-CT, lactose recrystallization

## Abstract

Caking represents a critical stability challenge for whey permeate powders (WPPs), frequently developing during storage and handling due to moisture-driven structural transformations within the powder bed. This study investigated the physical, morphological, and microstructural characteristics associated with caking in a limited set of industrial WPPs. Five commercial WPP samples differing in production date and storage conditions were characterized in terms of dry matter content, water activity (*a_w_*), particle size distribution (PSD), bulk density, porosity, color, and X-ray micro-computed tomography (micro-CT). Dry matter contents were similar among samples (97.74–98.20% *w.b.*); however, significant differences were observed in *a_w_*, bulk density, porosity, and PSD between the caked sample (WPP2) and the free-flowing powders. WPP2 exhibited the highest *a_w_* (0.261), the lowest bulk density (676 kg/m^3^), the highest porosity (0.569), and a distinctly coarser PSD. In addition, WPP2 showed the highest yellowness index (44.45), suggesting altered light-scattering behavior associated with structural changes. Micro-CT analysis revealed the presence of enlarged particle clusters and extensive particle–particle solid bridging in WPP2, accompanied by a heterogeneous pore distribution and reduced void connectivity, indicating consolidation of the powder bed. The integrated analytical approach demonstrates the potential of combining conventional measurements with micro-CT to provide detailed insight into the relationships between moisture-related properties and internal powder structure.

## 1. Introduction

Dairy-based powders, as an essential source of nutrition and calcium, represent high-value derivatives obtained from both primary dairy streams, such as skim milk, and co-products of cheese and butter manufacturing [1,2]. These powders serve as indispensable components in contemporary food products, with extensive application in infant nutrition, bakery and confectionery matrices, formulated beverages, sports nutrition, and specialized clinical formulations [3,4]. The production of such ingredients typically involves advanced fractionation technologies, most notably membrane filtration processes enabling selective partitioning of proteins, lactose, and mineral fractions. Following separation, concentrated streams undergo thermal treatment, predominantly through spray drying [5,6]. These unit operations are essential for reducing water activity (*a_w_*), enhancing microbial stability, prolonging shelf life, and improving storage and transport efficiency [6].

Whey permeate is a primary co-product of cheese manufacturing, generated through the ultrafiltration, nanofiltration, and electrodialysis of liquid whey to partition proteins and other high-molecular-weight fractions [7]. The manufacturing of whey permeate powder (WPP) involves controlled concentration of liquid permeate, induced crystallization of lactose, and subsequent spray drying of the crystallized slurry, followed by secondary drying to achieve a final moisture content of approximately 4–5% (*w.b.*) [8]. The resulting powder is characterized by high solubility, a neutral to mildly sweet dairy flavor profile [9], and a composition dominated by lactose (70–85% (*w*/*w*) of the dry solids) [7], with protein, residual minerals, and low-molecular-weight nitrogenous compounds [10,11]. From a techno-functional perspective, WPP functions as a bulking agent, mild sweetener, and stable carrier matrix in bakery and confectionery and as a versatile fermentation substrate in biotechnological processes [12].

Despite its industrial importance, WPP is highly prone to caking, a phenomenon widely recognized as a critical quality and processing challenge in dairy-based powders. Caking refers to the transformation of a free-flowing particulate material into cohesive or consolidated agglomerates during storage or handling, resulting in impaired flowability, reduced reconstitution performance, and significant economic losses [13]. The susceptibility of lactose-rich powders to caking has been extensively documented. Fitzpatrick et al. [14] demonstrated that the glass transition temperature (Tg) plays a key role in predicting stickiness and consolidation in dairy-based powders, while Roos [15,16] established the relationship between moisture sorption, Tg depression, and structural collapse in amorphous lactose matrices. More recent studies highlight the high sensitivity of lactose-rich powders to environmental humidity due to the hygroscopic nature of residual amorphous lactose [8,17].

At a mechanistic level, caking in WPPs is governed by a combination of moisture sorption and redistribution, capillary condensation within inter-particle voids, and lactose recrystallization kinetics [13,18]. Water acts as a plasticizer, and under conditions associated with reduced Tg, increased molecular mobility may promote viscous flow at particle contact points, facilitating capillary and solid bridge formation. Subsequent lactose crystallization can further consolidate the powder bed [19,20].

Numerous analytical approaches have been employed to characterize caking propensity in dairy-based powders. These include *a_w_* measurement and moisture sorption isotherms [17], differential scanning calorimetry for determining Tg and stickiness thresholds [8,14], particle size distribution and morphology analysis [21,22], and dynamic flowability and stickiness testing under controlled humidity conditions [23]. While these techniques provide valuable thermodynamic and kinetic insights, they primarily yield bulk-averaged parameters and may not adequately capture the early-stage structural evolution within the powder bed.

The literature increasingly acknowledges that caking initiation occurs at micro- and meso-scales through localized moisture accumulation, pore network connectivity, and particle–particle interactions preceding macroscopic consolidation [24,25]. However, direct visualization of these internal structural changes in lactose-rich powders remains limited. X-ray micro-computed tomography (micro-CT) represents a highly advanced, non-destructive imaging technique capable of resolving internal powder architecture in three dimensions at micro-scale resolution [26]. The potential of micro-CT to characterize food structures has been demonstrated in structurally engineered systems, such as extrusion-based 3D-printed protein- and dietary fiber-rich snacks composed of milk powder and wholegrain rye flour [27].

Although micro-CT has been previously employed to investigate agglomeration phenomena and structural features in selected powder materials [28], its application to diagnosing caking behavior in WPPs remains unexplored. The present study addresses this gap by proposing an integrated analytical approach for the identification of caking behavior in a limited set of industrial WPP samples, comprising five materials, including one exhibiting visible caking. Accordingly, the research aims to provide a detailed, case-specific assessment of the three-dimensional internal morphology associated with caking using micro-CT. By correlating moisture-related and physical parameters with quantitative structural descriptors, this work further aims to provide a deeper mechanistic understanding of caking occurrence, thereby contributing to improved quality control strategies and storage design in WPPs.

## 2. Results and Discussion

### 2.1. Physical Assessments

#### 2.1.1. Dry Matter (DM)

**Figure 1:**
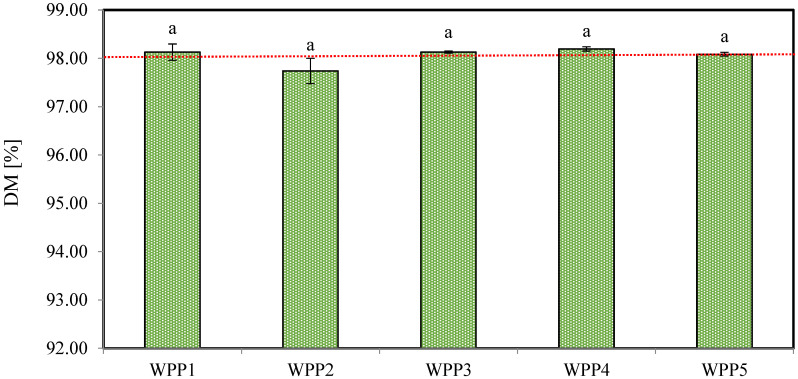
Dry matter (DM, %) content of the analyzed whey permeate powders (WPPs). Different lowercase letters on the bars indicate statistically significant differences among samples (Tukey’s test, *p* < 0.05). Error bars represent the standard deviation of three independent experiments.

Moisture acts as a plasticizer in lactose-rich powders such as WPPs and facilitates capillary condensation at particle contact points, promoting liquid bridge formation that may subsequently transform into solid bridges upon partial drying, a key mechanism underlying irreversible caking [29,30]. Figure 1 presents the dry matter (DM) content of the analyzed WPPs. The DM content ranged narrowly from 97.74 ± 0.26% (WPP2) to 98.20 ± 0.04% (WPP4), corresponding to moisture contents of approximately 1.81–2.26% (*w.b.*). Statistical analysis indicated no significant differences among samples (*p >* 0.05). However, sample WPP2, the only material exhibiting visible caking, showed the lowest DM content (97.74 ± 0.26%) and thus the highest moisture content (2.26%, *w.b.*).

Critical moisture thresholds in lactose-dominant powders are not absolute values but depend strongly on the interaction between moisture content, *a_w_*, and storage condition [31]. Roos [32] demonstrated that in amorphous lactose matrices, moisture contents approaching or slightly exceeding ~2% (*w.b.*) can significantly depress the Tg, particularly when accompanied by additional humidity sorption. In the present study, sample WPP2 exhibited a moisture content of 2.26% (*w.b.*), which falls within the range reported in the literature [32], where moisture-induced Tg depression may occur. Under such conditions, the physical state of WPP2 may have approached one associated with a reduced Tg, potentially leading to increased molecular mobility within the powder matrix. This behavior is commonly linked to enhanced stickiness and a higher susceptibility to caking in lactose-rich powders.

Although all powders were below the broader industrial moisture specification (≤4–5%) [8], sample WPP2 exhibited the highest moisture content among the analyzed WPPs and was the only material showing visible caking. Importantly, the difference between 2.26% (*w.b.*) (WPP2) and approximately 1.81–1.92% (*w.b.*) (other samples) may appear numerically small, yet previous studies indicate that moisture increments as low as 0.2–0.5% can markedly accelerate caking kinetics in lactose-containing powders [33]. Given the non-linear relationship between moisture and Tg depression [34], even this minor increase may have been sufficient to shift the effective Tg of sample WPP2 below the prevailing storage temperature, particularly if transient exposure to elevated relative humidity occurred.

Considering storage history, WPP2 had been stored for a relatively shorter period (97 days) compared with the other samples, which were stored for 103–388 days under controlled warehouse conditions. Despite this shorter storage duration, WPP2 exhibited caking. This observation indicates that storage duration alone was not the determining factor governing powder stability. WPP2 had been stored under uncontrolled environmental conditions, where fluctuations in ambient relative humidity and temperature could have occurred.

Notably, no consistent decrease in dry matter content was observed with increasing storage duration, as samples stored for extended periods (e.g., WPP4 for 388 days) maintained relatively high dry matter (≥ 98.08%). Therefore, while the moisture content of sample WPP2 remained below general industrial limits, it likely approached a critical stability window in which minor additional moisture uptake during storage, localized humidity gradients, or temperature fluctuations could have triggered plasticization, capillary bridge formation, and subsequent solid bridge consolidation.

#### 2.1.2. Water Activity (*a_w_*)

Understanding the water relations of WPPs is essential for optimizing processing conditions in industrial manufacturing and for controlling powder stability during transport and storage. Such an approach enables the identification of critical moisture content and water activity thresholds required to minimize caking. The storage stability of dairy-based powders is generally more strongly influenced by water activity than by moisture content [8,35].

**Figure 2:**
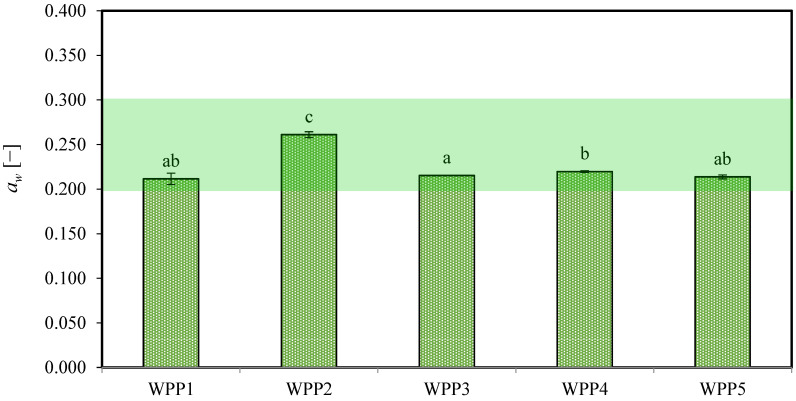
The water activity (*a_w_*) values of the analyzed whey permeate powders (WPPs). Different lowercase letters on the bars indicate statistically significant differences among samples (Tukey’s test, *p* < 0.05). Error bars represent the standard deviation of three independent experiments.

Figure 2 presents the *a_w_* values of the analyzed WPPs. In contrast to the relatively narrow range observed for dry matter content, *a_w_* showed statistically significant differences among samples (*p* < 0.05), ranging from approximately 0.212 ± 0.006 (WPP1) to 0.261 ± 0.003 (WPP2). Notably, sample WPP2 exhibited the highest *a_w_* (0.261 ± 0.003), indicating that a greater fraction of water was thermodynamically available rather than strongly bound within the powder matrix.

At low moisture levels, water is primarily associated with specific polar sites on lactose and proteins; however, as these sites approach saturation, even small increases in moisture content can result in a disproportionate rise in *a_w_* [36]. In the present study, the slightly higher moisture content observed in WPP2 (2.26%, *w.b.*), compared with the remaining samples, likely shifted the powder toward a region of the sorption isotherm characterized by increased water mobility. Consequently, the additional moisture not only increased total water content but also contributed to enhanced molecular mobility, as reflected in the elevated *a_w_*.

The remaining powders exhibited *a_w_* values between approximately 0.212 ± 0.006 and 0.220 ± 0.001, a range generally considered favorable for maintaining stability in lactose-rich powders under ambient conditions [37,38]. At *a_w_* < 0.23–0.25, amorphous lactose is typically reported to remain in a glassy state with limited molecular mobility and reduced susceptibility to stickiness and caking [32,37].

An increase in *a_w_* from approximately 0.212 ± 0.006 to 0.261 ± 0.003 has been associated in the literature with a reduction in Tg, potentially by 10–20 °C depending on composition and moisture distribution [39]. Under such conditions, the higher *a_w_* observed in WPP2 may have approached a range associated with increased molecular mobility. However, given the relatively small differences in *a_w_* among the samples, this parameter alone is unlikely to fully explain the observed caking behavior.

Within the analyzed samples, no consistent relationship between storage duration and aw was observed. In contrast, sample WPP2 exhibited the highest *a_w_* despite a shorter storage period (97 days compared with 103–388 days), suggesting that storage time alone was not the determining factor influencing thermodynamic water availability. Exposure to uncontrolled environmental conditions, including fluctuations in relative humidity and temperature, may have promoted moisture sorption and local redistribution within the powder bed, leading to an increase in *a_w_*. Therefore, in this study, the observed caking behavior is more likely the result of combined effects, including water activity, local moisture redistribution, storage conditions, and transient environmental exposure, which together may enhance molecular mobility and inter-particle interactions.

#### 2.1.3. Bulk Density (*ρ_b_*)

Bulk density, also referred to as packing density, is a key technological parameter governing the functional performance, economic efficiency, and storage stability of dairy-based powders. It reflects the combined effects of particle size distribution, morphology, surface composition, internal porosity, and interparticle packing efficiency [40,41]. In lactose-rich powders such as WPPs, bulk density is particularly relevant because it influences particle contact frequency, capillary condensation phenomena, and the susceptibility of amorphous lactose to moisture-induced phase transitions [42].

**Figure 3:**
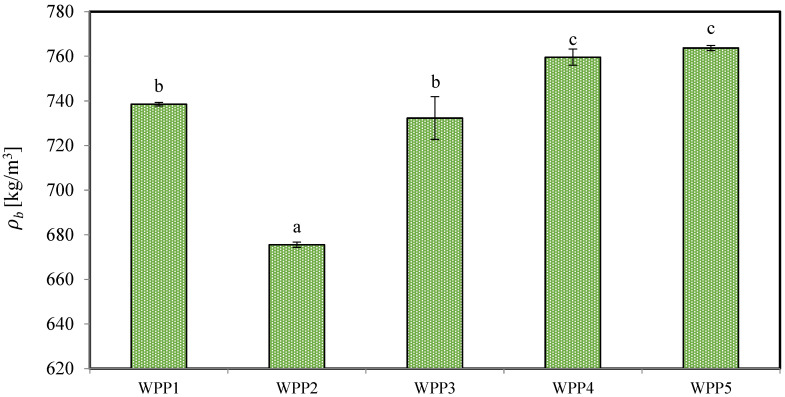
The bulk density (*ρ_b_*, kg/m^3^) values of the analyzed whey permeate powders (WPPs). Different lowercase letters on the bars indicate statistically significant differences among samples (Tukey’s test, *p* < 0.05). Error bars represent the standard deviation of three independent experiments.

The bulk density of the analyzed WPPs is presented in Figure 3. Values ranged from approximately 675.50 ± 1.17 kg/m^3^ (WPP2) to 763.71 ± 1.11 kg/m^3^ (WPP5), with statistically significant differences among samples (*p* < 0.05). The lowest bulk density was observed for WPP2. This observation is consistent with the elevated moisture content (2.26%, *w.b.*) and higher water activity (0.261 ± 0.003) observed in this sample, which may have contributed to increased molecular mobility and localized structural rearrangements within the powder bed. Such effects may promote the formation of a more open packing structure, thereby increasing interparticle void volume and reducing bulk density.

Notably, in this study, storage duration did not exhibit a clear relationship with bulk density. For example, WPP4 (storage period of 388 days) and WPP5 (storage period of 297 days), both stored under controlled warehouse conditions, exhibited the highest bulk densities (759.59 ± 3.62 and 763.71 ± 1.11 kg/m^3^, respectively), despite their longer storage times. This indicates that prolonged storage alone did not promote structural destabilization when environmental conditions were properly controlled.

Lodygin et al. [43] reported an average loose bulk density of approximately 791 kg/m^3^ for WPPs exhibiting no visible caking. The samples with the highest bulk density values (WPP4 and WPP5) approached this range and remained free-flowing, suggesting that controlled storage conditions can limit structural relaxation even during extended storage periods. In contrast, the markedly lower bulk density of WPP2, despite its shorter storage period (97 days), is likely associated with its exposure to uncontrolled environmental conditions. Fluctuations in ambient humidity and temperature may have promoted moisture sorption and subsequent structural rearrangement within the powder bed.

The bulk density results are consistent with the particle size distribution characteristics of WPP2 (Section Particle Size Distribution (PSD)). This sample exhibited the highest D[4,3] and D[3,2] values, indicating a coarser particle population and a reduced proportion of fine particles. Under loose packing conditions, coarse and relatively uniform particles pack less efficiently because fewer small particles are available to occupy interstitial voids. Consequently, interparticle void volume increases and bulk density decreases. However, in the case of WPP2, the elevated particle size parameters likely reflect storage-induced agglomeration rather than inherently larger primary particles. Overall, the results indicate that environmental storage conditions and moisture-driven structural evolution exerted a stronger influence on bulk density than storage duration alone.

#### 2.1.4. Porosity (ε)

In lactose-rich dairy-based powders, porosity plays a decisive role in determining moisture migration pathways, capillary condensation behavior, and the physical stability of amorphous lactose during storage [16,44].

**Figure 4:**
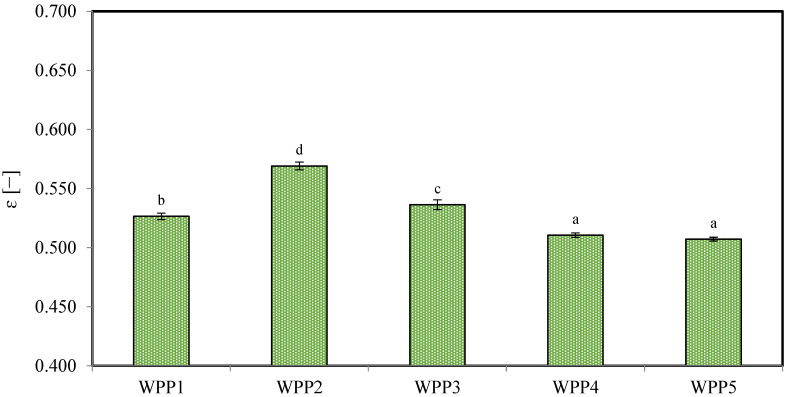
The porosity (ε) values of the analyzed whey permeate powders (WPPs). Different lowercase letters on the bars indicate statistically significant differences among samples (Tukey’s test, *p* < 0.05). Error bars represent the standard deviation of three independent experiments.

In the present study, porosity values ranged from approximately 0.507 ± 0.002 to 0.569 ± 0.003 and differed significantly among samples (*p* < 0.05) (Figure 4). Sample WPP2 exhibited the highest porosity (0.569 ± 0.003), which coincided with its lowest bulk density (675.50 ± 1.17 kg/m^3^), indicating a loosely packed structure with substantial void space. This structural configuration is likely a consequence of moisture absorption under uncontrolled storage conditions, where fluctuations in ambient relative humidity promoted water uptake by the hygroscopic powder. The resulting plasticization of the amorphous lactose phase may have facilitated particle adhesion and agglomeration. Such agglomeration increases internal void volume and disrupts efficient particle packing, leading to simultaneously higher porosity and lower bulk density.

In contrast, samples WPP5 (0.507 ± 0.002) and WPP4 (0.511 ± 0.002) exhibited the lowest porosity values and the highest bulk densities, reflecting dense particle packing and minimal interstitial air volume. These powders demonstrate efficient gravitational settling and limited internal pore connectivity. A reduced void fraction may restrict internal moisture redistribution pathways and may diminish the probability of localized capillary condensation. When accompanied by relatively high dry matter levels, this structural condition enhances mechanical rigidity and reduces susceptibility to moisture-induced plasticization and lactose recrystallization. Consequently, these samples exhibited higher storage stability and no observable caking.

#### 2.1.5. Color Specifications

Color is an important quality attribute of WPPs, reflecting compositional characteristics, thermal history, and potential physicochemical transformations occurring during processing and storage [45]. The CIE Lab* domain provides a standardized framework for evaluating lightness (L*), red–green chromaticity (a*), and yellow–blue chromaticity (b*), while the Whiteness Index (WI) and Yellowness Index (YI) provide integrated indicators of overall visual appearance. In lactose-rich dairy-based powders, color variations are typically influenced by lactose crystallinity, mineral composition, residual moisture, and the extent of non-enzymatic browning reactions, particularly Maillard reactions occurring during drying or storage [37,46].

The color specifications of the analyzed WPPs are presented in Table 1. The powders exhibited L* values ranging from approximately 70.04 ± 0.16 to 73.57 ± 0.09. Among the samples, WPP2 displayed the highest L* value (73.57 ± 0.09), suggesting a relatively brighter appearance compared with the other powders, whereas WPP4 exhibited the lowest L* value (70.04 ± 0.16), corresponding to a slightly darker tone. The relatively higher lightness observed for WPP2 may be associated with increased light scattering caused by surface irregularities or loosely bound agglomerates formed during caking.

All samples showed negative a* values (−4.23 ± 0.02 to −5.34 ± 0.01), confirming a slight shift toward the green region. The magnitude of variation in a* among samples was relatively small, indicating limited formation of Maillard-derived reddish pigments. WPP1 exhibited the most negative a* value (−5.34 ± 0.01), while WPP5 showed the least negative value (−4.23 ± 0.02), indicating a marginal shift toward the red direction in the latter. The relatively narrow range of a* values suggests that thermal browning reactions during processing or storage were minimal across all analyzed powders.

The b* parameter, representing yellowness, ranged from approximately 20.10 ± 0.10 to 22.89 ± 0.14. Sample WPP2 exhibited the highest b* value (22.89 ± 0.14), indicating a more pronounced yellow tone, whereas WPP4 showed the lowest value (20.10 ± 0.10). Increased yellowness in dairy-based powders may be associated with minor progression of Maillard reactions, differences in mineral composition, or variations in lactose crystallization behavior. Since whey permeate contains relatively low protein, extensive Maillard browning is unlikely; therefore, the observed variation in b* is more plausibly linked to compositional and structural differences rather than advanced thermal degradation [21]. In the case of WPP2, the higher b* value may also reflect changes in surface morphology or agglomeration caused by moisture-induced caking, which can alter light-scattering properties and enhance perceived yellowness.

The Whiteness Index (WI) ranged between 63.58 ± 0.004 and 65.89 ± 0.01, confirming the generally high whiteness typical of permeate powders. WPP3 (65.89 ± 0.01) and WPP5 (65.64 ± 0.32) exhibited the highest WI values, corresponding to relatively bright and optically uniform powders, whereas WPP1 and WPP4 showed slightly lower values. The Yellowness Index (YI) varied from approximately 39.53 ± 0.04 (WPP3) to 44.45 ± 0.23 (WPP2), further confirming the more pronounced yellow coloration of sample WPP2.

Overall, although color differences among the powders were relatively moderate, the higher L*, b*, and YI values observed for WPP2 may reflect structural and physicochemical changes associated with moisture-induced agglomeration and caking. Such structural modifications can influence light-scattering behavior at particle surfaces, leading to subtle yet measurable variations in optical properties compared with the free-flowing powders.

**Figure 5:**
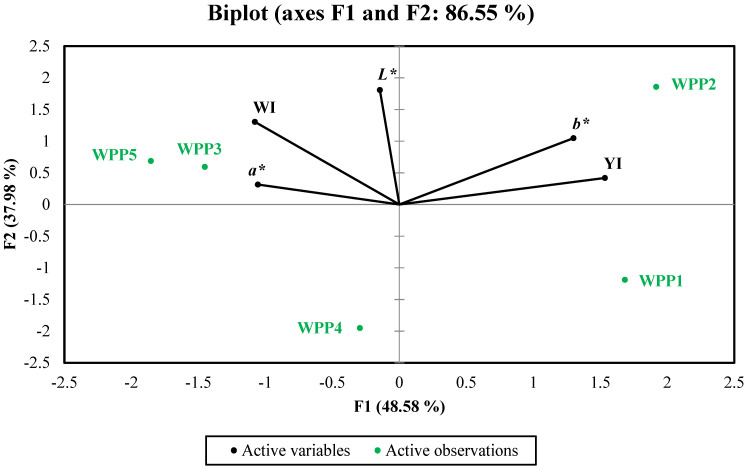
PCA biplot of color specifications (L*, a*, b*, WI, and YI) of analyzed whey permeate powders (WPPs). L*: lightness; a*: greenness; b*: yellowness; WI: Whiteness Index; YI: Yellowness Index.

The Principal Component Analysis (PCA) biplot (Figure 5) revealed a clear separation of WPP samples based on their color characteristics, with the first two principal components explaining 86.55% of the total variance. The first principal component (PC1, 48.58%) was primarily associated with yellowness-related parameters (b* and YI), while the second component (PC2, 37.98%) was mainly influenced by lightness (L*) and, to a lesser extent, the Whiteness Index (WI).

Sample WPP2 was distinctly separated along the positive PC1 axis and strongly aligned with b* and YI, confirming its higher yellowness compared with the other samples. This observation is consistent with its elevated yellowness index and suggests that caking-induced structural changes, such as agglomeration and altered surface morphology, influenced light-scattering behavior and enhanced the perceived yellow coloration.

In contrast, WPP3 and WPP5 were located on the negative side of PC1 and showed closer association with WI and a*, indicating more typical color characteristics of free-flowing lactose-rich powders. These samples exhibited higher whiteness and more uniform optical properties, reflecting a stable microstructure and limited moisture-induced alterations.

WPP1, although positioned on the positive PC1 axis, was separated along the negative PC2 direction, indicating a different combination of color attributes and a weaker association with lightness compared with WPP2. Meanwhile, WPP4 was located in the negative PC2 region with limited alignment to any specific variable, suggesting less pronounced differentiation in color attributes among the analyzed parameters.

Overall, the PCA confirms that the caked sample (WPP2) is primarily distinguished by increased yellowness rather than changes in lightness, supporting the hypothesis that moisture-induced structural modifications influence the optical properties of whey permeate powders.

### 2.2. Morphological Analysis

#### Particle Size Distribution (PSD)

**Figure 6:**
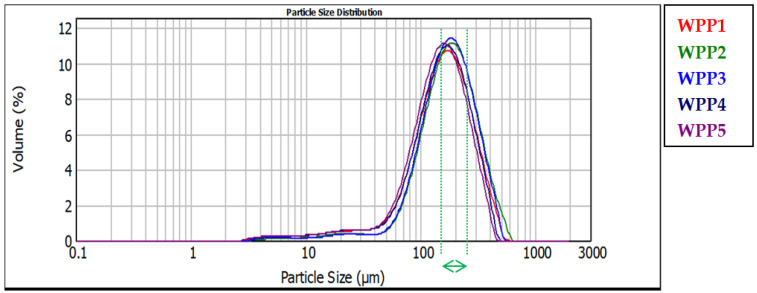
Volume-based particle size distribution curves of the analyzed whey permeate powders (WPPs) determined by laser diffraction.

The PSD curves of the analyzed WPPs are presented in Figure 6. All samples exhibited a predominantly unimodal distribution with a main peak located between approximately 140 and 180 μm. This range is in agreement with the median mass diameter (d(0.5)) reported in Table 2 for each sample. Although the overall shape of the curves is similar, slight horizontal shifts in the peak position and differences in distribution width indicate variability in the particle population among WPPs. In particular, the curve corresponding to WPP2 (green line) is visibly shifted toward larger particle diameters, confirming a coarser particle distribution compared to the other powders. Conversely, WPP4 and WPP5 show peaks positioned at slightly smaller diameters, reflecting finer particle populations.

Table 2 presents particle size distribution parameters of analyzed WPPs, including volume-weighted mean diameter (D[4,3]), surface-weighted mean diameter (D[3,2]), and percentile diameters at 10%, 50%, and 90% cumulative volume distribution (d(0.1), d(0.5), and d(0.9)), respectively. Notable differences are revealed among the WPPs, reflecting variations in particle population characteristics and structural organization. The D[4,3] ranged from 159.87 ± 3.31 µm (WPP5) to 197.61 ± 3.96 µm (WPP2), indicating that WPP2 possessed the coarsest overall particle population. A similar trend was observed for the D[3,2], which varied from 81.11 ± 0.42 µm (WPP5) to 115.31 ± 4.75 µm (WPP2), yet with WPP2 exhibiting the highest value. The elevated D[3,2] in WPP2 suggests a reduced proportion of fine particles relative to the other samples, which may influence both packing behavior and surface-driven interactions such as moisture sorption. Rennie et al. [47] reported that dairy-based powders with 20 μm particles were twice as cohesive as the 40 μm particles. The electrostatic force holding particles together also influences the reconstitution properties, including wettability, sinkability and dispersibility. Very fine particles do not wet readily because of their high surface tension and small inter-particle space [41]. Therefore, the powder particles need to be large enough and of uniform size to flow easily and dissolve rapidly during reconstitution.

The median particle diameter, d(0.5), ranged between 147.87 ± 2.89 µm (WPP5) and 178.71 ± 3.51 µm (WPP2), while the coarse-tail parameter, (d(0.9), extended from 279.16 ± 7.16 µm (WPP5) to 343.55 ± 6.55 µm (WPP2). Sample WPP2 displayed the highest d(0.5) and d(0.9) values, confirming a right-shifted distribution with a pronounced coarse fraction. In contrast, WPP5 exhibited the smallest characteristic diameters across all percentiles, indicating a relatively finer and more compact distribution. The span between d(0.1) and d(0.9) suggests that all samples present moderately broad distributions, yet WPP2 appears slightly more polydisperse.

Importantly, the elevated particle size parameters observed for WPP2 likely do not reflect the primary particle formation process during processing. Given that all batches were produced under comparable industrial conditions. This is more plausibly attributed to storage-induced consolidation and caking. Laser diffraction analysis disperses particles mechanically but does not fully eliminate strongly bonded agglomerates formed via solid bridge formation. Therefore, the larger D[4,3], D[3,2], d(0.1), d(0.5), and d(0.9) values likely reflect the presence of partially consolidated clusters generated during storage rather than intrinsically larger primary particles in WPP2.

Sample WPP2 had been stored under uncontrolled environmental conditions prior to analysis. Under such uncontrolled conditions, fluctuations in ambient relative humidity and temperature may occur, which can significantly influence the hygroscopic behavior of powders. Exposure to elevated humidity can promote gradual moisture sorption by the powder particles, while temperature variations may further enhance moisture redistribution within the powder bed. Consequently, these moisture-driven agglomeration phenomena may lead to the formation of loosely bound secondary particle clusters, which are subsequently detected as larger particle sizes in laser diffraction measurements. Therefore, the increased particle size observed for WPP2 may reflect moisture-induced agglomeration occurring during uncontrolled storage rather than inherent differences in the primary particle structure.

From a functional perspective, PSD strongly influences bulk density, porosity, and caking propensity [48]. Coarser particles typically promote lower packing efficiency when accompanied by irregular morphology or weak agglomerate structures, potentially increasing the void fraction [49]. Overall, the PSD results indicate that sample WPP2 is characterized by a coarser and potentially more agglomerated structure due to visible caking.

### 2.3. Micro-Computed Tomography (Micro-CT) Analysis of WPPs’ Structure

#### 2.3.1. Visual Assessment of Internal Powder Structure

**Figure 7:**
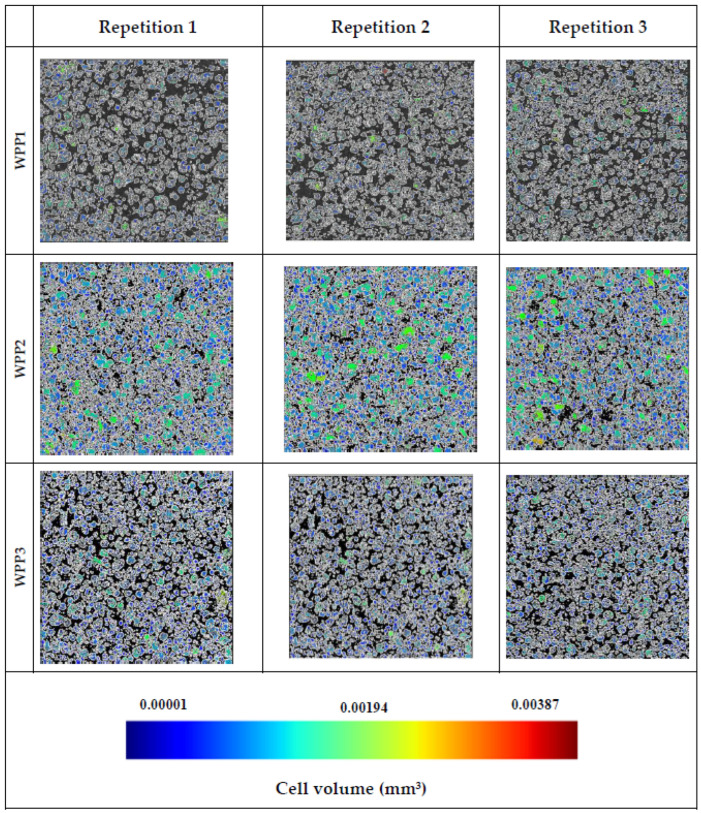
Representative micro-computed tomography (micro-CT) cross-sections of independent regions of interest for WPP1, WPP2, and WPP3.

**Figure 8:**
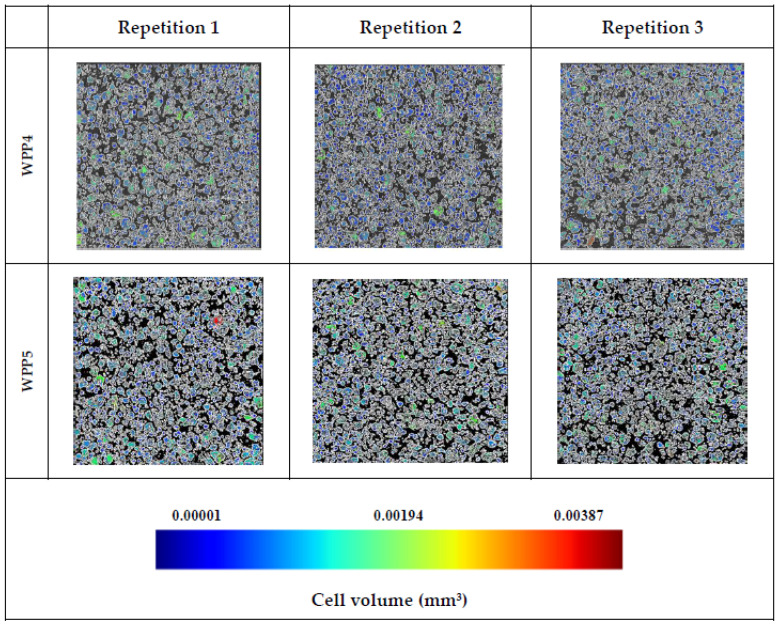
Representative micro-computed tomography (micro-CT) cross-sections of independent regions of interest for WPP4 and WPP5.

Representative micro-CT cross-sections of the analyzed WPPs are presented in Figure 7 and Figure 8. Clear and reproducible differences in internal packing structure, pore architecture, and solid-phase connectivity were observed among the samples. For each WPP, three independent regions of interest (repetitions 1–3) were evaluated, confirming the consistency and representativeness of the detected microstructural features.

The free-flowing powders (WPP1, WPP3, WPP4, and WPP5) exhibited microstructures dominated by discrete, well-defined particles embedded within a continuous and interconnected void phase. Particle boundaries were sharply distinguishable, and inter-particle contacts were predominantly limited to point contacts or small contact areas characteristic of non-caked powders. The inter-particle pore network was relatively homogeneous, with numerous small- and medium-sized voids evenly distributed throughout the powder bed.

In particular, WPP4 and WPP5, which exhibited the highest bulk densities and lowest porosity values, showed a more compact spatial arrangement of particles, reflected by smaller and more uniformly distributed interstitial pores. Their comparatively finer particle size distributions (lower D[4,3], D[3,2], d(0.5), and d(0.9) values) likely facilitated partial filling of interstitial voids by smaller particles, thereby enhancing packing efficiency and reducing the presence of large interparticle pores.

In contrast, for sample WPP2, across all repetitions, micro-CT cross-sections revealed extensive solid-phase connectivity between adjacent particles. Instead of discrete spherical entities, multiple particles were fused through continuous solid bridges, forming irregular agglomerated clusters with partially blurred or indistinct inter-particle boundaries. This confirms that the coarse particle size distribution observed by laser diffraction reflects storage-induced agglomeration rather than intrinsic particle formation during processing.

These microstructural findings are coherent with the previously discussed physical and morphological parameters. The coarser PSD of WPP2 (highest D[4,3] and D[3,2]) reduced the availability of fine particles to occupy interstitial spaces, predisposing the powder bed to larger inter-particle voids. Concurrently, the slightly elevated moisture content (2.26%) and significantly higher water activity (0.261 ± 0.003) likely promoted localized plasticization of amorphous lactose. Under such conditions, viscous flow at particle contacts may facilitate capillary bridge formation. Upon moisture redistribution or partial drying, these liquid bridges may solidify into permanent solid bridges, producing the continuous solid-phase connectivity observed by micro-CT. The color-coded cell-volume maps indicate that WPP2 contained a higher proportion of green and yellow regions corresponding to higher cell volumes. In contrast, the remaining powders were characterized predominantly by blue regions, suggesting a more uniform particle size distribution.

Importantly, the consolidation observed in WPP2 is not uniform compaction but a heterogeneous structural transformation characterized by clustered connectivity and irregular pore enlargement. The reproducibility of these features across repetitions confirms that the structural reorganization is intrinsic to the material and not a superficial artifact of sampling. Overall, the micro-CT results provide direct three-dimensional evidence that caking in WPP2 arises from moisture-mediated inter-particle bonding and heterogeneous pore restructuring, linking particle size distribution, bulk density reduction, increased porosity, and elevated water activity into a coherent structural mechanism.

Furthermore, the absence of pronounced color differences among samples suggests that the structural changes observed in WPP2 are primarily associated with moisture-induced physical consolidation rather than advanced browning or chemical degradation. Overall, the micro-CT analysis provides direct three-dimensional evidence linking inter-particle bridging and altered pore architecture to the combined effects of particle size distribution, water activity, bulk density, and porosity. These findings demonstrate that microstructural connectivity constitutes a critical structural indicator of caking progression in lactose-dominant WPPs.

#### 2.3.2. Object Volume Distributions Derived from Micro-CT

**Figure 9:**
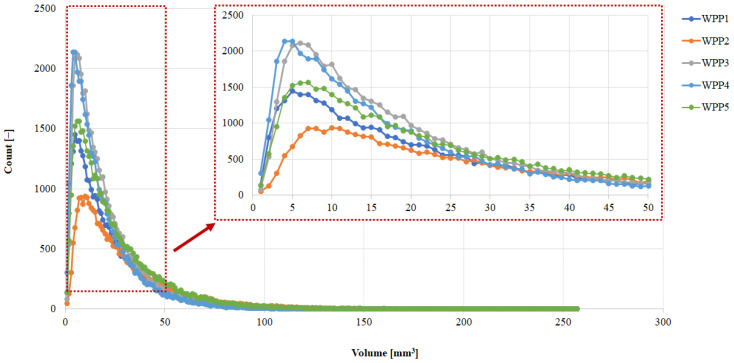
Object volume distributions of whey permeate powders (WPPs) derived from connected-component analysis of segmented micro-CT datasets.

To quantitatively characterize the microstructural differences observed in the micro-CT cross-sections, frequency distributions of connected solid object volumes were derived from connected-component analysis of the segmented solid phase. The resulting object volume distributions are presented in Figure 9.

For the free-flowing powders (WPP1, WPP3, WPP4, and WPP5), the distributions are strongly right-skewed, with a dominant peak in the low-volume region and a rapid monotonic decrease toward larger volumes. This pattern indicates that most solid entities exist as discrete particles or very small, weakly connected clusters within the powder bed. Although all samples exhibit a long tail extending toward higher volumes, the frequency of large connected components remains limited, consistent with predominantly point-contact interactions and the absence of extensive solid bridging. These findings align with the previously reported higher bulk densities and more homogeneous pore networks, reflecting efficient yet non-consolidated packing.

In contrast, sample WPP2 exhibits a distinctly modified object volume distribution profile. While small objects are still present, their relative frequency is reduced compared to the other samples, and the distribution displays a clear rightward extension, indicating an increased contribution of larger connected components. The more gradual decay and amplified tail in the high-volume region reflect the presence of multi-particle clusters formed through inter-particle bridging and structural coalescence. This redistribution of object volumes quantitatively confirms the enhanced solid-phase connectivity visually identified in the micro-CT cross-sections.

The inset focusing on the low-volume region (0–50 mm^3^) further highlights the divergence of WPP2 from the remaining powders. Even at small structural scales, this sample exhibits reduced counts of isolated particles and a higher proportion of intermediate-sized clusters, indicating that consolidation initiated at the microstructural level before progressing to larger agglomerates. These quantitative results are fully consistent with the physicochemical data: the higher water activity, slightly lower dry matter, coarser particle size distribution, and reduced bulk density of WPP2 collectively promoted inter-particle plasticization and bridge formation. Thus, the object volume analysis provides robust three-dimensional evidence linking moisture-driven physicochemical instability to the development of internal solid-phase connectivity and caking in WPPs.

#### 2.3.3. Structural Interpretation in the Context of Caking

The micro-CT-derived morphological parameters summarized in Table 3 reveal measurable differences in the average particle-equivalent diameter, surface area, and volume among the analyzed WPP samples. Most powders exhibited comparable values, with equivalent diameters ranging from approximately 0.0729 ± 0.0040 to 0.0743 ± 0.0045 mm and average particle volumes between (2.50 ± 0.58) × 10^−4^ and (2.78 ± 0.65) × 10^−4^ mm^3^, indicating relatively uniform particle populations. In contrast, sample WPP2, which exhibited visible caking, showed noticeably higher values for all three parameters, with an equivalent diameter of 0.0878 ± 0.0078 mm, an average surface area of 0.0911 ± 0.0165 mm^2^, and an average particle volume of (4.35 ± 1.45) × 10^−4^ mm^3^. These increases suggest the presence of larger connected particle structures rather than individual primary particles. The results therefore support the interpretation that the apparent particle enlargement detected by micro-CT reflects storage-induced agglomeration and inter-particle bridging associated with caking. This observation is consistent with the PSD results and the microstructural images, which indicated extensive solid-phase connectivity and cluster formation in WPP2 compared with the discrete particle packing observed in the free-flowing samples.

The combined qualitative (Figure 7 and Figure 8) and quantitative (Figure 9) micro-CT results demonstrate that caking in WPPs is associated with a distinct transition from a microstructure dominated by discrete particles to one characterized by extensive solid-phase connectivity. Visually, this transition manifests as the loss of clearly defined particle boundaries and the formation of continuous solid bridges between neighboring particles (Figure 7 and Figure 8). Quantitatively, this process is reflected in a redistribution of solid object volumes toward fewer and larger connected entities (Figure 9). Importantly, these micro-CT-derived structural descriptors should be interpreted as markers of particle connectivity and agglomeration rather than as direct measures of individual particle size. The observed increase in the frequency and volume of large connected objects indicates the formation of inter-particle bridges and consolidation within the native packed powder bed. Notably, these structural signatures of caking were observed despite relatively similar particle size distributions determined by laser diffraction. This confirms that caking-related structural evolution cannot be inferred from dispersed particle size measurements alone, but instead arises from particle–particle interactions, moisture redistribution, and phase transitions occurring within the intact powder bed. The micro-CT analysis therefore provides direct three-dimensional evidence of internal structural consolidation underlying the macroscopic caking behavior observed in sample WPP2.

### 2.4. Correlation

**Figure 10:**
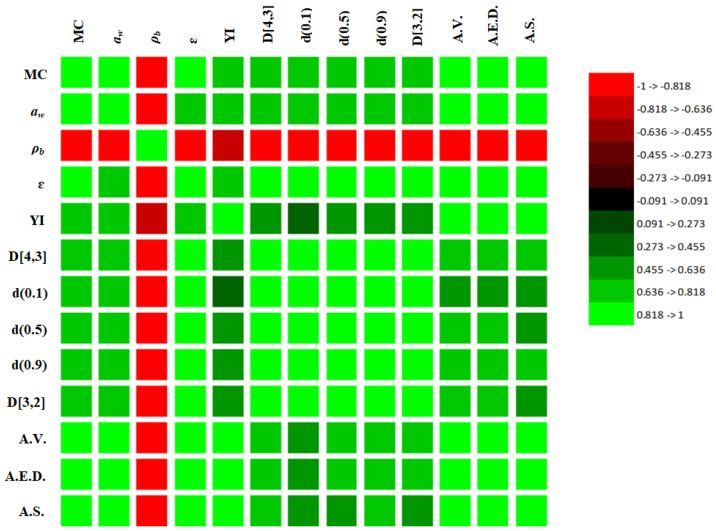
Heatmap showing Pearson correlation coefficients (r) among physical, morphological, and micro-CT parameters. MC: moisture content; YI: yellowness index; A.V.: average volume, A.E.D.: average equivalent diameter; A.S.: average surface.

The relationships among the physical, morphological, and microstructural properties of the analyzed WPPs were evaluated using Pearson correlation analysis. The heatmap of correlation coefficients (*r*), the correlation matrix, and the corresponding matrix of *p*-values are presented in Figure 10, Table 4, and Table 5, respectively. Overall, the results revealed strong and statistically significant interdependencies among the parameters obtained from physical measurements, particle size distribution, and micro-CT structural characterization, indicating that the macroscopic physical behavior of the powders is strongly governed by their internal structural organization.

A strong positive correlation was observed between moisture content (MC) and water activity (*a_w_*) (*r* = 0.942, *p* = 0.017), confirming that an increase in MC contributes directly to higher thermodynamic water availability in the powder. Both MC and *a_w_* were also strongly correlated with the structural parameters derived from micro-CT analysis. In particular, MC exhibited very strong positive correlations with average particle volume (A.V.) (*r* = 0.963, *p* = 0.008), average equivalent diameter (A.E.D.) (*r* = 0.967, *p* = 0.007), and average surface area (A.S.) (*r* = 0.937, *p* = 0.019). These relationships indicate that increased moisture availability is associated with larger connected particle structures, supporting the interpretation that moisture-induced agglomeration and inter-particle bonding occurred in the powder bed.

Water activity showed a strong negative correlation with bulk density (*ρ****_b_***) (*r* = −0.894, *p* = 0.040) and a moderate, though not statistically significant (*p* > 0.05), positive relationship with porosity (ε) (*r* = 0.848, *p* = 0.069), indicating that increased thermodynamic water availability is associated with reduced packing efficiency and a tendency toward more open structures. This behavior is consistent with the observed characteristics of WPP2, where elevated *a_w_* coincided with lower bulk density, higher porosity, and larger apparent particle sizes.

Bulk density showed strong negative correlations with most structural and particle size parameters. In particular, significant negative correlations were observed between *ρ****_b_*** and D[4,3] (r = −0.896, p = 0.040), d(0.5) (r = −0.866, p = 0.058), d(0.9) (r = −0.914, p = 0.030), and D[3,2] (r = −0.884, p = 0.046). Similar inverse relationships were also observed between *ρ****_b_*** and the micro-CT structural descriptors A.V., A.E.D., and A.S. (r = −0.903 to −0.917, *p* < 0.05). These correlations suggest that powders composed of larger particles or agglomerated clusters tend to pack less efficiently, resulting in lower bulk density and greater internal void space.

Color properties also showed meaningful associations with structural parameters. The yellowness index (YI) exhibited positive correlations with several particle size parameters and micro-CT descriptors (e.g., YI–A.S.: *r* = 0.928, *p* = 0.023), suggesting that powders with larger structural dimensions tend to exhibit higher YI. This behavior may be attributed to differences in light scattering and surface morphology associated with particle enlargement and agglomeration.

Furthermore, particle size descriptors showed positive relationships with the micro-CT-derived structural parameters. For example, D[3,2] exhibited positive correlations with A.V. (*r* = 0.642, *p* = 0.230) and A.E.D. (*r* = 0.664, *p* = 0.221), indicating a directionally consistent trend, though these relationships did not reach statistical significance at *p* < 0.05.

Overall, the correlation analysis demonstrates a strong coupling between moisture availability, particle size distribution, packing efficiency, and internal structural architecture in whey permeate powders. However, given the limited sample size, these relationships should be interpreted as indicative of trends within the analyzed dataset rather than as broadly generalizable behavior. Importantly, the strong agreement between conventional particle size measurements and micro-CT structural descriptors confirms that the use of these integrated analytical techniques provides a comprehensive understanding of the physical and microstructural mechanisms governing powder stability.

## 3. Materials and Methods

### 3.1. Materials

#### Specifications of the WPPs Provided by the Company

Commercial food-grade WPPs, non-hydrolyzed and non-instant, were supplied by an industrial dairy processor for the characterization of caked powder through integrated physical, morphological, and microstructural analyses. To investigate the characteristics of both caked and non-caked powders, five representative samples were selected (Table 6). The corresponding batches were manufactured under comparable industrial processing conditions and formulations. The primary differences among the batches concerned the production date and storage conditions. This selection strategy enabled the assessment of storage-related effects on powder stability while minimizing variability associated with processing parameters.

Among the analyzed samples, WPP2 exhibited visible signs of caking and was therefore considered representative of a powder exhibiting advanced structural consolidation. In contrast, the remaining WPPs (WPP1, WPP3, WPP4, and WPP5) did not exhibit visible caking at the time of sampling. According to the producer, these non-caked powders had been stored under controlled warehouse conditions, with temperatures ranging from 18 to 25 °C and relative humidity between approximately 30 and 50%, depending on seasonal variations. In contrast, sample WPP2 had been maintained under uncontrolled environmental conditions prior to analysis. Upon receipt from the producer, all WPP samples were transferred into airtight polypropylene containers and immediately subjected to physical, morphological, and structural analyses to minimize potential alterations in moisture content and powder structure during handling.

### 3.2. Methods

#### 3.2.1. Physical Assessments

##### Dry Matter (DM)

The dry matter content of the samples was determined according to the international standard ISO 5537|IDF 26:2023 [50], which specifies a gravimetric oven-drying procedure for dried milk and milk-based products. The milk powders were dried at 87 °C for 5 h in a laboratory dryer SLW 53 (POL-EKO^®^, Wodzisław Śląski, Poland). After drying, the samples were cooled in a desiccator to room temperature and then weighed. Dry matter content was then calculated by mass difference and reported on a wet basis (*w.b.*). Moisture content was calculated as the complementary fraction and likewise reported on a wet basis (*w.b*.). All determinations were performed in triplicate for each sample.

##### Water Activity (*a_w_*)

The water activity of the analyzed samples was measured using an AQUALAB Series 4TEV water activity meter (AQUALAB, Warsaw, Poland). Measurements were performed at a temperature of 25 °C. All determinations were performed in triplicate for each sample.

##### Bulk Density (*ρ_b_*)

Bulk density was determined in accordance with PN-ISO 8460:1999 [51] and defined as the ratio of the mass of loosely poured WPP to the known volume of the cylinder. The measurement was performed by allowing the powder to flow freely through a funnel positioned axially above a cylindrical container with a volume of 65 cm^3^. The powder was poured without mechanical compaction to ensure loose filling conditions. Excess material was carefully removed by leveling the surface with a glass plate to obtain a flat, reproducible surface flush with the container edge. The filled container was then weighed using an analytical balance (AS 220/C/2, Radwag, Radom, Poland). Bulk density was then calculated as the mass of WPP divided by the container volume.

(1) $$\rho_{b}=\frac{m_{p}}{v_{c}}$$

where $\rho_{b}$ is the bulk density, $m_{p}$ is the mass of WPP sample (kg), and $v_{c}$ is the volume of the cylindrical container (m^3^).

##### Pycnometric Density ($\rho_{p}$)

The pycnometric density was determined using an automatic gas pycnometer (HumiPyc II, InstruQuest Inc., Coconut Creek, FL, USA). Measurements were performed in an argon atmosphere at a relative pressure of 220 kPa to ensure accurate determination of the true solid volume by gas displacement. Prior to analysis, samples were conditioned to laboratory temperature to minimize thermal expansion effects. The instrument calculated the sample volume based on pressure changes between the reference and measurement chambers. Pycnometric density was subsequently determined as the ratio of the measured sample mass to the true volume obtained from the gas displacement measurement.

(2) $$\rho_{p}=\frac{m_{p}}{v_{p}}$$

where $\rho_{p}$ is pycnometric density (kg/m^3^), $m_{p}$ is the mass of the WPP sample (kg), and $v_{p}$ is the volume of the powder (m^3^).

##### Porosity (ε)

The porosity of the WPPs was determined indirectly from the experimentally measured bulk density and pycnometric density values. Porosity was defined as the fraction of void volume within the bulk powder bed relative to the total volume occupied by the sample. This parameter was calculated based on the difference between the true density of the solid material, obtained by gas pycnometry, and the bulk density measured under loose filling conditions. The resulting porosity (ε) therefore reflects the combined contribution of inter-particle voids and accessible intra-particle pores within the powder structure and was calculated according to Equation (3).

(3) $$\varepsilon =1-\frac{\rho_{b}}{\rho_{p}}$$

where ε is the porosity, $\rho_{b}$ is the bulk density (kg/m^3^) and $\rho_{p}$ is the pycnometric density (kg/m^3^).

##### Color Specifications

The color parameters in the CIE Lab* color space were determined using a colorimetric method with a CR-400/410 colorimeter (Konica Minolta, Tokyo, Japan), operated in conjunction with Spectra Magic™ NX software version 2.0 for data acquisition and processing. Measurements were performed using the standard illuminant D65 and a 2° standard observer angle, in accordance with the recommendations of the International Commission on Illumination (CIE).

##### Whiteness Index (WI)

The WI of the WPP samples was calculated through Equation (4) [52].

(4) $$WI=100-\sqrt{{\left(100-L^{*}\right)}^{2}+{a^{*}}^{2}+{b^{*}}^{2}}$$

##### Yellowness Index (YI)

The YI of the WPP samples was calculated through Equation (5) [53].

(5) $$YI=142.86\times \frac{b^{*}}{L^{*}}$$

#### 3.2.2. Morphological Analysis

##### Particle Size Distribution

The particle size distribution was measured using a Mastersizer 2000 laser particle analyzer (Malvern Instruments Ltd., Malvern, Worcestershire, UK) in dry mode with the Sirocco 2000 accessory, using the laser diffraction technique in full accordance with ISO 13320-1 [54].

#### 3.2.3. Structural Analysis by X-Ray Micro-Computed Tomography (Micro-CT)

##### Micro-CT System and Scanning Conditions

Micro-computed tomography (micro-CT) measurements were performed using a phoenix V|tome|x S X-ray microtomography device (Waygate Technologies, Hürth, Germany) equipped with a 240 kV microfocus X-ray tube. WPPs were scanned in their native, loose-packed state without any mechanical compaction to preserve the original packing structure.

**Figure 11:**
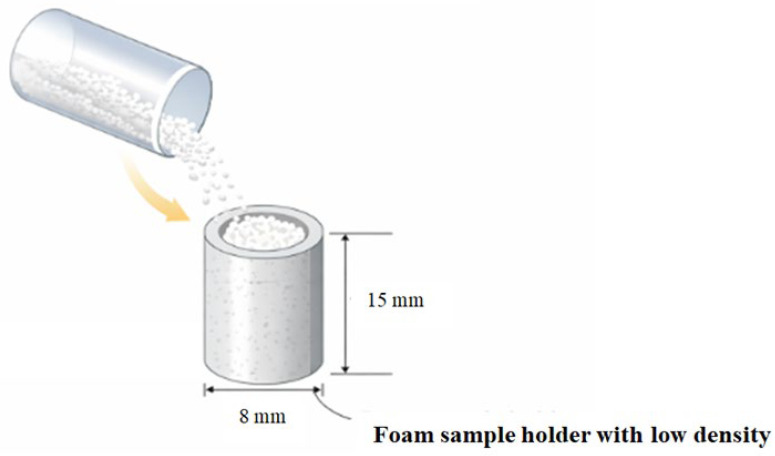
Schematic illustration of WPP filling into a low-density foam cylindrical holder prior to scanning.

The WPP samples were analyzed in their native powder form without prior mechanical treatment. For micro-computed tomography (micro-CT) analysis, the material was transferred into a cylindrical sample holder fabricated from low-density foam characterized by a low X-ray attenuation coefficient. The selection of a low-absorbing support material minimized beam hardening and imaging artefacts while ensuring mechanical stability of the sample during scanning. The holder had an internal diameter of 8 mm, an external diameter of 12 mm, and a height of 15 mm (Figure 11). The powder was gently poured into the holder under gravity and subsequently placed in the micro-CT scanner chamber. No controlled compaction, vibration, or tapping protocol was applied, thereby preserving the native packing structure of the powder bed for structural analysis.

##### X-Ray Acquisition Parameters

**Figure 12:**
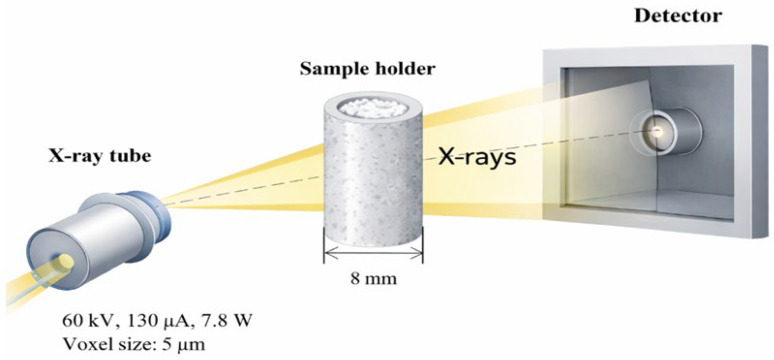
Schematic representation of the X-ray micro-CT setup used in this study.

The samples were scanned using X-ray micro-computed tomography under operating conditions of a 60 kV acceleration voltage and a 130 µA tube current, corresponding to a power output of 7.8 W. Data acquisition was performed with skip set to 1 and frame averaging set to 2, while the exposure time for a single projection was 200 ms. The scanning procedure was conducted at a spatial resolution corresponding to a voxel size of 5 µm. A schematic representation of the measurement setup, including the X-ray source–sample–detector configuration, is presented in Figure 12.

##### Reconstruction and Region-of-Interest Selection

Reconstruction of the raw projection data was carried out using phoenix datos|x software version 2022 (Waygate Technologies, Hürth, Germany) employing a filtered back-projection algorithm. A motion correction routine was applied during reconstruction to reduce noise and artefacts associated with potential sample vibration or minor displacement during scanning. The reconstructed volumes were stored in 16-bit grayscale format. To ensure comparability among samples and to exclude the low-density foam holder from further analysis, a cubic region of interest (ROI) of 500 × 500 × 500 voxels was extracted from the central part of each reconstructed volume. This ROI selection minimized edge effects and ensured that only the internal powder structure was analyzed. The voxel size of 5 µm defines the resolution limit of the analysis, meaning that structural features smaller than approximately 2–3 voxels (i.e., 10–15 µm) may not be reliably resolved. This limitation is particularly relevant for very fine pores and thin inter-particle bridges, which may be partially affected by voxel averaging. Potential imaging artefacts include partial volume effects, beam hardening, and noise-related fluctuations in grayscale intensity. Partial volume effects may occur at phase boundaries, leading to intermediate grayscale values and affecting segmentation accuracy. Beam hardening was minimized through the use of a low-density sample holder and relatively low acceleration voltage. Additionally, motion correction during reconstruction and central ROI selection were applied to further reduce artefact influence.

##### Segmentation of the Solid Phase

Segmentation of the solid phase was performed in phoenix datos|x using an isovalue-based automatic thresholding approach. The threshold separating the solid powder material from the void phase was determined automatically by the software for each dataset based on the grayscale intensity distribution. The automatic threshold selection implemented in phoenix datos|x is based on the analysis of the grayscale histogram, identifying the transition between low-density (air/void) and high-density (solid material) phases. The selected threshold corresponds to the inflection region between the two peaks in the bimodal intensity distribution, ensuring a data-driven and consistent phase separation across samples. The automatically selected threshold values typically fell within the range of approximately 19,000–25,000 grayscale units, reflecting variations in image contrast among samples. Voxels with grayscale values above the threshold were classified as solid material, while voxels below the threshold were assigned to the void phase. The applied thresholding procedure corresponds to a global histogram-based segmentation approach, in which a single threshold value was assigned to each dataset without local or adaptive thresholding. To ensure consistency and reliability of phase separation, the segmentation results were visually inspected for each dataset by comparing grayscale slices with the corresponding binary images. In addition, segmentation quality was verified by visual assessment to confirm accurate separation of solid and void phases and to ensure that particle boundaries and inter-particle contacts were physically meaningful. No additional morphological post-processing operations (such as erosion, dilation, or watershed-based particle separation) were applied. Consequently, direct particle–particle contacts were preserved in the segmented volumes, allowing the native connectivity and agglomeration state of the powder bed to be retained for subsequent structural analysis.

##### Structural Analysis and Object-Based Quantification

Quantitative structural analysis of the segmented solid phase was conducted using the powder analysis module in phoenix datos|x in material analysis mode. Connected-component labeling was applied to identify individual solid objects within the ROI, where an object was defined as a contiguous cluster of solid voxels. For each identified object, the object volume was calculated based on the voxel count and voxel size. Frequency distributions of object volumes were then constructed to evaluate changes in the size and connectivity of solid entities within the powder bed. These distributions were used as quantitative descriptors of structural coalescence and agglomeration associated with caking behavior. The object volume distributions were visualized as histograms expressing the number of solid objects as a function of their volume. Particular attention was paid to shifts in the distributions toward fewer, larger connected objects, which are indicative of particle coalescence and inter-particle bridging.

##### Interpretation of Micro-CT-Derived Structural Descriptors

The micro-CT-derived structural descriptors were interpreted as markers of structural connectivity and agglomeration rather than as direct measures of individual particle size. In this context, increases in the frequency and volume of large connected solid objects were associated with the formation of inter-particle bridges and the loss of discrete particle morphology, characteristics of caking in lactose-rich powders. The micro-CT results were evaluated in conjunction with physicochemical parameters, including dry matter content, water activity, bulk density, porosity, and particle size distribution, to establish a mechanistic link between moisture-related changes and the observed structural evolution of the powder bed. Despite the resolution limitations inherent to micro-CT analysis, the applied methodology provides robust comparative structural descriptors, as all samples were analyzed under identical scanning, reconstruction, and segmentation conditions.

##### Software and Powder Analysis Workflow

All reconstructed micro-CT datasets were processed and analyzed using VGStudio MAX software version 2022 (Volume Graphics GmbH, Heidelberg, Germany). Structural analysis of the powder bed was performed using the dedicated Powder Analysis workflow implemented in VGStudio MAX. Following segmentation of the solid phase, connected-component labeling was applied to identify individual solid objects, defined as contiguous clusters of solid voxels. Object connectivity was determined based on voxel face connectivity and no artificial particle separation or watershed-based splitting was applied. This approach ensured that physically connected particles remained classified as single objects, preserving the native agglomeration and consolidation state of the powder bed. For each identified object, the volume was calculated directly from the voxel count and voxel size. Object volume distributions were subsequently extracted and used as quantitative descriptors of structural connectivity and agglomeration within the powder bed. Visualization of object volumes was performed by color-coding connected objects according to their volume, enabling both two-dimensional cross-sectional inspection and three-dimensional rendering of the internal powder structure.

### 3.3. Statistical Analysis

All measurements were performed in triplicate for each WPP sample. The results are presented as means ± standard deviations (SDs). Statistical analyses were performed using Minitab^®^ Statistical Software, version 19.1.10. Differences among samples were evaluated by one-way analysis of variance (one-way ANOVA) at a significance level of *p* < 0.05. When the ANOVA indicated significant effects, mean comparisons were conducted using Tukey’s post hoc multiple comparison test. Pearson correlation coefficients (r) and the corresponding *p*-values were calculated to assess relationships among selected physical, morphological, and microstructural variables using XLSTAT, version 2025.1.1. Principal component analysis (PCA) was also performed in XLSTAT to explore multivariate patterns and variability within the dataset.

## 4. Conclusions

This study investigated the physical, morphological, and microstructural characteristics of five industrial WPPs, including one sample exhibiting visible caking. Within this limited sample set, the caked powder (WPP2) was characterized by lower dry matter, higher water activity, lower bulk density, higher porosity, and a larger apparent particle size compared to the free-flowing powders (WPP1, WPP3, WPP4, and WPP5). Although the analyzed samples exhibited only minor differences in dry matter content, statistically significant and technologically relevant variations were observed in water activity, particle size distribution, and the internal pore structure of the caked and non-caked powders. These differences were reflected in pronounced changes in internal solid-phase connectivity, as revealed by X-ray micro-CT. WPP2 exhibited a coarser apparent particle size distribution and extensive inter-particle solid bridging, indicating a transition from a discrete-particle matrix to a more interconnected structural network. In contrast, WPP1, WPP3, WPP4, and WPP5 maintained distinct particle boundaries and a more homogeneous pore structure. The results further indicate that differences in storage conditions may have contributed to the observed behavior. While the non-caked samples were stored under controlled warehouse conditions, WPP2 had been exposed to uncontrolled environmental conditions prior to analysis, where fluctuations in relative humidity and temperature could have occurred. Such exposure may have promoted moisture sorption, increased water activity, and facilitated structural transformations associated with caking. It should be emphasized that the present findings are based on a limited number of industrial samples, including a single caked powder, and therefore should be interpreted as case-specific observations rather than generalized behavior for all WPPs. Nevertheless, the integrated analytical approach provided detailed insight into the relationships among moisture availability, particle size distribution, and internal structural evolution within the analyzed powders. Overall, this study demonstrates the applicability of X-ray micro-CT as a powerful tool for the qualitative and quantitative characterization of powder microstructure, enabling the assessment of particle connectivity, pore architecture, and structural heterogeneity in lactose-rich matrices.

## Figures and Tables

**Table 1:** Color specifications of the analyzed whey permeate powders in the CIE Lab* domain.

Sample Code	L*	a*	b*	WI	YI
WPP1	70.87 ± 0.02 ^b^	−5.34 ± 0.01 ^g^	21.20 ± 0.02 ^b^	63.58 ± 0.004 ^a^	42.74 ± 0.03 ^d^
WPP2	73.57 ± 0.09 ^d^	−4.84 ± 0.04 ^d^	22.89 ± 0.14 ^c^	64.70 ± 0.05 ^b^	44.45 ± 0.23 ^e^
WPP3	72.95 ± 0.01 ^c^	−4.95 ± 0.02 ^e^	20.18 ± 0.02 ^a^	65.89 ± 0.01 ^c^	39.53 ± 0.04 ^a^
WPP4	70.04 ± 0.16 ^a^	−4.58 ± 0.02 ^c^	20.10 ± 0.10 ^a^	63.63 ± 0.08 ^a^	40.99 ± 0.13 ^c^
WPP5	72.67 ± 0.44 ^b–d^	−4.23 ± 0.02 ^b^	20.39 ± 0.06 ^a^	65.64 ± 0.32 ^bc^	40.08 ± 0.13 ^b^

WPP: whey permeate powder. Different lowercase superscript letters within a column indicate statistically significant differences among samples (Tukey’s Test, *p* < 0.05).

**Table 2:** Particle size distribution parameters of analyzed whey permeate powders (WPPs).

Sample Code	D[4,3]	d(0.1)	d(0.5)	d(0.9)	D[3,2]
WPP1	174.99 ± 1.31 ^b^	65.27 ± 0.58 ^b^	159.25 ± 0.79 ^b^	310.73 ± 2.93 ^c^	89.39 ± 1.31 ^b^
WPP2	197.61 ± 3.96 ^c^	83.21 ± 2.51 ^c^	178.71 ± 3.51 ^a^	343.55 ± 6.55 ^e^	115.31 ± 4.75 ^c^
WPP3	190.19 ± 6.03 ^c^	82.49 ± 3.29 ^c^	174.34 ± 5.61 ^c^	326.98 ± 9.95 ^d^	109.28 ± 3.34 ^c^
WPP4	168.93 ± 1.47 ^b^	63.04 ± 1.41 ^ab^	156.53 ± 1.29 ^b^	296.42 ± 2.05 ^b^	82.77 ± 1.69 ^a^
WPP5	159.87 ± 3.31 ^a^	61.75 ± 0.34 ^a^	147.87 ± 2.89 ^a^	279.16 ± 7.16 ^a^	81.11 ± 0.42 ^a^

D[4,3]: volume-weighted mean diameter; D[3,2]: surface-weighted mean diameter; d(0.1), d(0.5), d(0.9): particle diameters at 10%, 50%, and 90% cumulative volume distribution, respectively. Different lowercase superscript letters within a column indicate statistically significant differences among samples (Tukey’s Test, *p* < 0.05).

**Table 3:** Micro-CT-derived morphological parameters of analyzed whey permeate powders (WPPs).

Sample Code	Avg. Equivalent Diameter [mm]	Avg. Surface [mm^2^]	Avg. Volume [mm^3^]
WPP1	0.0743 ± 0.0045	0.0620 ± 0.0085	(2.78 ± 0.65) × 10^−4^
WPP2	0.0878 ± 0.0078	0.0911 ± 0.0165	(4.35 ± 1.45) × 10^−4^
WPP3	0.0729 ± 0.0040	0.0499 ± 0.0072	(2.50 ± 0.58) × 10^−4^
WPP4	0.0739 ± 0.0043	0.0544 ± 0.0078	(2.66 ± 0.62) × 10^−4^
WPP5	0.0734 ± 0.0042	0.0528 ± 0.0075	(2.61 ± 0.60) × 10^−4^

Avg.: average.

**Table 4:** Matrix of Pearson correlation coefficients (r) among variables obtained from physical, morphological, and micro-CT analyses of WPPs.

Variables	MC	*a_w_*	*ρ_b_*	ε	YI	D[4,3]	d(0.1)	d(0.5)	d(0.9)	D[3,2]	A.V.	A.E.D.	A.S.
MC	**1**	**0.942**	**−0.907**	0.863	0.752	0.657	0.619	0.620	0.665	0.695	**0.963**	**0.967**	**0.937**
*a_w_*	**0.942**	**1**	**−0.894**	0.848	0.760	0.688	0.618	0.667	0.696	0.683	**0.976**	**0.985**	**0.936**
$\rho_{b}$	**−0.907**	**−0.894**	**1**	**−0.993**	−0.766	**−0.896**	−0.815	−0.866	**−0.914**	**−0.884**	**−0.912**	**−0.917**	**−0.903**
ε	0.863	0.848	**−0.993**	**1**	0.699	**0.940**	0.874	**0.915**	**0.950**	**0.932**	0.857	0.865	0.846
YI	0.752	0.760	−0.766	0.699	**1**	0.482	0.268	0.423	0.571	0.397	0.868	0.842	**0.928**
D[4,3]	0.657	0.688	**−0.896**	**0.940**	0.482	**1**	**0.959**	**0.997**	**0.992**	**0.980**	0.656	0.673	0.633
d(0.1)	0.619	0.618	−0.815	0.874	0.268	**0.959**	**1**	**0.968**	**0.916**	**0.990**	0.552	0.581	0.499
d(0.5)	0.620	0.667	−0.866	**0.915**	0.423	**0.997**	**0.968**	**1**	**0.980**	**0.979**	0.618	0.640	0.587
d(0.9)	0.665	0.696	**−0.914**	**0.950**	0.571	**0.992**	**0.916**	**0.980**	**1**	**0.953**	0.687	0.698	0.681
D[3,2]	0.695	0.683	**−0.884**	**0.932**	0.397	**0.980**	**0.990**	**0.979**	**0.953**	**1**	0.642	0.664	0.602
A.V.	**0.963**	**0.976**	**−0.912**	0.857	0.868	0.656	0.552	0.618	0.687	0.642	**1**	**0.999**	**0.989**
A.E.D.	**0.967**	**0.985**	**−0.917**	0.865	0.842	0.673	0.581	0.640	0.698	0.664	**0.999**	**1**	**0.981**
A.S.	**0.937**	**0.936**	**−0.903**	0.846	**0.928**	0.633	0.499	0.587	0.681	0.602	**0.989**	**0.981**	**1**

MC: moisture content; YI: yellowness index, A.V.: average volume, A.E.D.: average equivalent diameter; A.S.: average surface. Values in bold are different from 0 with a significance level alpha = 0.05.

**Table 5:** Matrix of *p*-values for Pearson correlation coefficients among variables obtained from physical, morphological, and micro-CT analyses of WPPs.

Variables	MC	*a_w_*	*ρ_b_*	ε	YI	D[4,3]	d(0.1)	d(0.5)	d(0.9)	D[3,2]	A.V.	A.E.D.	A.S.
MC	**0**	**0.017**	**0.034**	0.059	0.142	0.229	0.265	0.264	0.220	0.193	**0.008**	**0.007**	**0.019**
*a_w_*	**0.017**	**0**	**0.040**	0.069	0.136	0.199	0.267	0.219	0.191	0.204	**0.005**	**0.002**	**0.019**
$\rho_{b}$	**0.034**	**0.040**	**0**	**0.001**	0.131	**0.040**	0.093	0.058	**0.030**	**0.046**	**0.031**	**0.028**	**0.035**
ε	0.059	0.069	**0.001**	**0**	0.189	**0.018**	0.053	**0.029**	**0.013**	**0.021**	0.063	0.058	0.071
YI	0.142	0.136	0.131	0.189	**0**	0.411	0.663	0.478	0.314	0.508	0.056	0.073	**0.023**
D[4,3]	0.229	0.199	**0.040**	**0.018**	0.411	**0**	**0.010**	**0.000**	**0.001**	**0.003**	0.230	0.213	0.252
d(0.1)	0.265	0.267	0.093	0.053	0.663	**0.010**	**0**	**0.007**	**0.029**	**0.001**	0.334	0.305	0.392
d(0.5)	0.264	0.219	0.058	**0.029**	0.478	**0.000**	**0.007**	**0**	**0.003**	**0.004**	0.266	0.245	0.298
d(0.9)	0.220	0.191	**0.030**	**0.013**	0.314	**0.001**	**0.029**	**0.003**	**0**	**0.012**	0.200	0.190	0.206
D[3,2]	0.193	0.204	**0.046**	**0.021**	0.508	**0.003**	**0.001**	**0.004**	**0.012**	**0**	0.243	0.221	0.282
A.V.	**0.008**	**0.005**	**0.031**	0.063	0.056	0.230	0.334	0.266	0.200	0.243	**0**	**<0.0001**	**0.001**
A.E.D.	**0.007**	**0.002**	**0.028**	0.058	0.073	0.213	0.305	0.245	0.190	0.221	**<0.0001**	**0**	**0.003**
A.S.	**0.019**	**0.019**	**0.035**	0.071	**0.023**	0.252	0.392	0.298	0.206	0.282	**0.001**	**0.003**	**0**

MC: moisture content; YI: yellowness index, A.V.: average volume, A.E.D.: average equivalent diameter; A.S.: average surface. Bold values indicate statistically significant correlations according to Pearson’s correlation analysis (*p* < 0.05).

**Table 6:** Specification of the whey permeate powders (WPPs) received from the producer.

Sample Code	Production Date	Caking Status	Storage Time (Day)
WPP1	28 August 2025	Non-caked	103
WPP2	3 September 2025	Caked	97
WPP3	23 July 2025	Non-caked	147
WPP4	24 November 2024	Non-caked	388
WPP5	23 February 2025	Non-caked	297

## Data Availability

Data are contained within the article.
